# Transcriptome data of *Epinephelus fuscoguttatus* infected by *Vibrio vulnificus*

**DOI:** 10.1016/j.dib.2017.11.024

**Published:** 2017-11-13

**Authors:** Fahmeeda Mohamad Jazamuddin, Wan Mohd Aizat, Hoe-Han Goh, Chen-Fei Low, Syarul Nataqain Baharum

**Affiliations:** Institute of Systems Biology (INBIOSIS), Universiti Kebangsaan Malaysia, UKM, Bangi 43600, Selangor, Malaysia

**Keywords:** Transcriptome, Grouper, Vibriosis, Epinephelus fuscoguttatus

## Abstract

Vibriosis disease by *Vibrio* spp. greatly reduced productivity of aquaculture, such as brown-marbled grouper (*Epinephelus fuscoguttatus*), which is an economically important fish species in Malaysia. Preventive measures and immediate treatment are critical to reduce the mortality of *E. fuscoguttatus* from vibriosis. To investigate the molecular mechanisms associated with immune response and host-bacteria interaction, a transcriptomic analysis was performed to compare between healthy and *Vibrio*-infected groupers. This permits the discovery of immune-related genes, specifically the resistance genes upon infection. Herein, we provide the raw transcriptome data from Illumina HiSeq. 4000 that have been deposited into NCBI SRA database with the BioProject accession number PRJNA396437. A total of 493,403,076 raw sequences of 74.5 Gb were obtained. Trimming of the raw data produced 437,186,232 clean reads of ~58 Gb. These datasets will be useful to elucidate the defence mechanisms of *E. fuscoguttatus* against *Vibrio vulnificus* infection for future development of effective prevention and treatment of vibriosis.

**Specifications Table**

**Table:** 

Subject area	Biology
More specific subject area	Transcriptomics
Type of data	Transcriptome sequences
How data was acquired	Illumina HiSeq. 4000
Data format	Raw data (FASTQ)
Experimental factors	Survival of groupers on day 31 post infection
Experimental features	Datasets for gill and whole-body tissues
Sample source location	Universiti Malaysia Terengganu (UMT), Terengganu, Malaysia (5°24'26.6"N 103°05'17.1"E)
Data accessibility	http://www.ncbi.nlm.nih.gov/bioproject/396437

**Value of the data**

• This dataset provides the transcriptome sequences from gill and whole-body tissues of control (healthy) and *Vibrio-*infected *E. fuscoguttatus*.
• Downstream analysis will allow the identification of genes involved in the mechanism of host immune response against *Vibrio* infection.
• This study will provide a better understanding on the molecular mechanisms associated with immune defense and host-pathogen interaction.
• The data can be a reference transcriptome for grouper and useful for comparative analysis with other fish diseases.

## 1 Data

This article reports the transcriptome data from gill and whole-body tissues of control (healthy) and *V. vulnificus-*infected *E. fuscoguttatus* at day 31 post infection. The raw data were deposited in the NCBI SRA database as detailed in Table 1.

**Table 1:** SRA accession links for brown-marbled group data.

**Sample Name**	**Biological replicates**	**Accession number**	**Accession links**
Control gill	CG-1	SRX3067297	http://www.ncbi.nlm.nih.gov/sra/SRX3067297
	CG-2	SRX3067296	http://www.ncbi.nlm.nih.gov/sra/SRX3067296
	CG-3	SRX3067301	http://www.ncbi.nlm.nih.gov/sra/SRX3067301
			
Infected gill	RG-1	SRX3067298	http://www.ncbi.nlm.nih.gov/sra/SRX3067298
	RG-2	SRX3067295	http://www.ncbi.nlm.nih.gov/sra/SRX3067295
	RG-3	SRX3067304	http://www.ncbi.nlm.nih.gov/sra/SRX3067304
			
Control whole-body	CWB-1	SRX3067303	http://www.ncbi.nlm.nih.gov/sra/SRX3067303
	CWB-2	SRX3067302	http://www.ncbi.nlm.nih.gov/sra/SRX3067302
	CWB-3	SRX3067300	http://www.ncbi.nlm.nih.gov/sra/SRX3067300
			
Infected whole-body	RWB-1	SRX3067299	http://www.ncbi.nlm.nih.gov/sra/SRX3067299
	RWB-2	SRX3067293	http://www.ncbi.nlm.nih.gov/sra/SRX3067293
	RWB-3	SRX3067294	http://www.ncbi.nlm.nih.gov/sra/SRX3067294

## 2 Experimental design, materials and methods

### 2.1 Fish sampling and infection experiment

Brown-marbled grouper (*Epinephelus fuscoguttatus*) fingerlings were obtained from Fisheries Research Institute (FRI) Tanjung Demong, Besut in Terengganu, Malaysia (5°46'09.0"N 102°33'02.6"E). Fingerlings with length of ~5 cm were acclimatised in aerated seawater (25 °C, salinity ~35 parts per thousand (PPT), pH 7.9) for one week. For experimental infection, healthy fingerlings were randomly selected and immersed in 20 L seawater containing 1×10^7^ CFU mL^−1^ of *Vibrio vulnificus* for 30 min, whereas the controls were immersed in clean seawater. Both control and infected fingerlings were then transferred independently into new aquarium containing fresh seawater, and observed daily for 30 days. At day 31-post-infection, fingerlings that survived from the experimental infection and the controls were collected and flash frozen in liquid nitrogen respectively. Samples were then kept at −80 °C prior to RNA extraction.

### 2.2 Total RNA Extraction and quality control, library preparation and RNA-seq

Total RNA was extracted from gills and whole-body tissues using modified hexadecyltrimethylammonium bromide (CTAB) method [1]. Ten biological replicates from each tissue were used for the RNA extraction. The quality and integrity of the isolated RNA were quantified using NanoDrop spectrophotometer (Thermo Fisher Scientific Inc., United States) and Agilent 2100 Bioanalyzer (Agilent Technologies, Inc., United States), respectively. Only three replicates from each tissue with the highest RIN score were chosen for RNA sequencing. The cDNA poly (A)-containing mRNA libraries were then prepared according to the SureSelect Strand-Specific RNA Library Prep for Illumina Multiplexed Sequencing (mRNA Library Preparation Protocol) Version E0, March 2017. Libraries were then sequenced using Illumina HiSeq. 4000 platform at Theragene Etex Bio Institute (Gyeonggi-do, Republic of Korea).

### 2.3 Transcriptome *de novo* assembly

Raw reads obtained were filtered by trimming the adapter sequences using cutadapt v1.14 program [2] with the Phred quality score of 30 (nucleotide accuracy: 99.9%) (Table 2). Clean reads were then *de novo* assembled using Trinity v2.4.0 [3].

**Table 2:** Statistics of gill and whole-body sequence reads.

**Sample**	**Pre-filter**		**Post-filter**	
	**Number of reads**	**Number of bases (bp)**	**Number of reads**	**Number of bases (bp)**
Control gill	123,109,176	18,589,485,576	113,060,376	14,771,245,333
Infected gill	116,155,918	17,539,543,618	100,270,060	13,137,845,799
Control whole-body	128,443,514	19,394,970,614	109,603,414	14,719,744,887
Infected whole-body	125,694,468	18,979,864,668	114,252,382	15,376,382,424
